# Electrocautery ablation therapy for anal intraepithelial carcinoma: A study protocol

**DOI:** 10.1097/MD.0000000000032297

**Published:** 2022-12-23

**Authors:** Naokatsu Ando, Daisuke Mizushima, Misao Takano, Hiroshi Kitamura, Daisuke Shiojiri, Takato Nakamoto, Takahiro Aoki, Koji Watanabe, Haruka Uemura, Hiroyuki Gatanaga, Shinichi Oka

**Affiliations:** a AIDS Clinical Center, National Center for Global Health and Medicine, Tokyo, Japan.

**Keywords:** anal cancer, anal intraepithelial neoplasia, electrocautery ablation, human papilloma virus, men who have sex with men

## Abstract

Anal cancer is a human papillomavirus-related cancer. Screening with high-resolution anoscopy (HRA) and subsequent therapeutic intervention are increasingly recognized as the standard procedure for anal cancer. Generally, lesions suspected as being high-grade squamous intraepithelial lesions are biopsied and treated if they are grade 2 or 3 anal intraepithelial neoplasia (AIN). According to several studies, electrocautery ablation for grade 2 or 3 AIN is highly effective. However, relapse within and outside the targeted areas after the intervention is a clinical problem. In Japan, electrocautery ablation is not available at most facilities. Therefore, this study aims to investigate the efficacy and safety of electrocautery ablation.

**Methods:**

This single-arm, open-label, pilot intervention study will investigate the efficacy and safety of electrocautery ablative therapy using high-frequency medical devices. Patients diagnosed with grade 2 or 3 AIN will be included and will receive ablation treatment. Then, they will be followed up at 3 and 6 months after the procedure for HRA-guided sextant biopsy. To reduce the possibility of missed lesions before and after the intervention, we will perform HRA-guided sextant biopsy routinely. In this study, a sextant biopsy is defined as at least 6 biopsies in all directions, regardless of abnormal findings under HRA. The primary outcome is the recurrence rate at 6 months, and the secondary outcomes are the adverse event and recurrence rates at 3 months.

**Conclusion:**

This pilot study will provide data on the effectiveness and safety of electrocautery ablation as a treatment for grade 2 or 3 AIN.

## 1. Introduction

Anal cancer is a human papillomavirus (HPV)-associated cancer. More than 90% of anal cancers are caused by HPV, and most of them are HPV16 and 18.^[1]^ The annual incidence of anal cancer in the general population is as low as 1.4 per 100,000 person-years but has remarkably increased by 2.7% per year over the decades.^[2]^ In addition, the at-risk population was reportedly people living with HIV (PLWH), men who have sex with men (MSM), women with HPV-related cancer or precancerous lesions, people with immunosuppression, and smokers.^[3]^ Therefore, a higher incidence of 84 per 100,000 person-years for HIV-positive MSM, which was the highest risk factor, was reported.^[3]^ The prevention of anal cancer is an essential part of anal cancer management because poor prognosis is associated with cancer diagnosis at a more advanced stage.^[4]^

Anal cancer is preceded by high-grade squamous intraepithelial lesion (HSIL), which comprises grade 2 or 3 anal intraepithelial neoplasia (AIN). HSIL in PLWH progresses to cancer at the rate of 402 per 100,000 person-years.^[5]^ At present, a screening program with anal cytology and HPV genotyping is recommended in some at-risk populations based on expert opinion.^[4,6,7]^ Recently, the ANCHOR study conducted in the United States with a large sample size demonstrated that the use of a screening program with interventions for PLWH with HSIL decreased the rate of progression to anal cancer by 57%.^[5]^ In this study, interventions included electrocautery ablation, infrared coagulation, excision, and topical agents, but electrocautery ablation was used most often (83.6%). Electrocautery ablation may be a primary method, as it is an easy, fast, and office-based procedure. Previous studies have shown that electrocautery ablation is effective for treating HSIL with few adverse events.^[5,8,9]^ However, relapse at approximately 1 year after the procedure is a primary concern. The rate of relapse is 25% to 61%; therefore, recurrent procedures may be required for relapsed lesions.^[8,10]^

In Japan, a screening program using high-resolution anoscopy (HRA) is not well established because HRA is available at only a few hospitals for research purposes. Moreover, no treatment options have been approved by the National Insurance for people with HSIL. However, screening with HRA showed that 73.1% (31/52) of participants had HSIL among PLWH, as a previous study showed.^[11]^ These screening programs, followed by effective interventions need to be introduced on priority.

Surgitron, is a popular medical device used for tonsillectomy, eye disease, and anorectal diseases.^[12–15]^ The device provides a high radio frequency for precise ablation that is effective only for targeted lesions and minimizes unintended invasion, leading to few side effects. However, there have been no studies on the treatment of HSIL using this device. Therefore, this pilot study aims to investigate the effectiveness and safety of electrocautery using the Surgitron dual EMC.

## 2. Methods

### 2.1. Ethics statements

This study was approved by the Certified Review Board of National Center for Global Health and Medicine (NCGM-C-004310-00). This trial was registered in the Japan Registry of Clinical Trials (Clinical Trial Plan Number: jRCTs032210649).

The current research protocol is version 1.1, dated October 21, 2021. The principal investigator will prepare an explanatory document, consent document, and consent withdrawal form, which shall be approved by a clinical research review committee approved by the NCGM. The consent document will be prepared for clinical research. The consent document must be prepared in plain language so that it can be understood by the participant in the clinical research (a person who is independent of the person who conducts the clinical research). Persons involved in this research will not obtain personal information through deception or other wrongful means, will make utmost efforts to protect the personal information and privacy of the clinical research participants, and will not divulge personal information obtained in the course of conducting this research without justifiable reason (the same shall apply even after the persons involved leave their positions). In addition, those involved in this research must not handle personal information obtained in the course of conducting the research beyond the scope of consent obtained in advance from the participants of the clinical research. When handling personal information, the principal investigator must specify the purpose of use as much as possible and must keep personal information accurate and up to date to the extent necessary to achieve the purpose of use. They must also take necessary measures to prevent leakage, loss, or damage of personal information and for other appropriate management of personal information, and must specifically stipulate the methods of such measures in the Implementation Rules.

### 2.2. Study design and population

The single-arm, open-label, pilot intervention will investigate the efficacy and safety of electrocautery ablative therapy with the Surgitron for patients with grade 2 or 3 AIN. The study will be conducted in the outpatient clinics of the AIDS Clinical Center and sexual health clinic at the National Center for Global Health and Medicine (NCGM). Staff at the AIDS Clinical Center will provide HIV-infected patients with medical care and treatment. Staff at the sexual health clinic will follow-up high-risk MSM without HIV and regularly assess their HIV status and other sexually transmitted infections. In total, 20 patients with grade 2 or grade 3 AIN will be included in the study for electrocautery ablation and follow-up biopsy with HRA. For sample size calculation to detect a safety problem, a pilot study sample size of 20 has a probability of at least 0.9 that a problem will occur in at least one of the participants. This assumes that each individual has a probability of 0.1 of exhibiting the problem.

#### 2.2.1. Participant recruitment

The participants will be recruited from the center hospital of the NCGM. The study will be explained to the participants by their primary physician, and they will be asked to sign a consent form.

#### 2.2.2. Inclusion criteria

Patients who meet the following criteria will be eligible to participate in the study.

1. Persons or their legal representatives who provide written consent for the participation of the patient in the trial.
2. Persons aged 20 years of age or older at the time consent was obtained.
3. Persons diagnosed with grade 2 or 3 AIN based on sextant biopsy results under HRA within 1 year. Sextant biopsy is defined as at least 6 samples in all directions, regardless of abnormal findings.

#### 2.2.3. Exclusion criteria

Patients who meet any of the following criteria will not be eligible to participate in the study.

1. Persons with invasive cancer.
2. Persons whom the principal investigator, investigator, or sub-investigator judge to be ineligible for other reasons.
3. Pregnant or breastfeeding women.

#### 2.2.4. Study protocol

Table 1 shows the study schedule. A physician will explain the study protocol to each candidate, and if consent is obtained, the physician will ask the candidate to sign an informed consent form and perform the examination in keeping with the description in Table 1. At visit 1, patients with AIN will undergo electrocautery ablation. At visits 2 and 3, a physician will confirm adverse events due to the intervention and perform HRA with a biopsy.

**Table 1:** Study schedule.

	Screening	Visit 1 (day 1)	Visit 2 (day 90)	Visit 3 (day 180)
			±45 days	± 45 days
Informed consent	x			
Confirmation of eligibility (inclusion and exclusion criteria)	x			
Enrollment	x			
Clinical characteristics	x			
Vital signs		x	x	x
Physical findings	x	x	x	x
Electrocautery ablation		x		
Adverse events		x	x	x
High-resolution anoscopy			x	x
Tissue biopsy			x	x

### 2.3. Intervention

#### 2.3.1. Electrocautery ablation

The patient will be placed in the left lateral recumbent position and an anoscope will be inserted to magnify the anal canal for colposcopy. Then, we will apply 3% acetic acid to observe the nebulous anal TZ (aceto-white lesion) surface, margins, and vascular abnormalities, and together with the results of HRA guidance, the ablation area for biopsy will be determined. The targeted area with 3 to 5-mm margins will be ablated with a 4-MHz radiofrequency device (Surgitron; Ellman Japan, Inc., Osaka, Japan). This device offers precise ablation of the targeted area and minimizes postprocedural pain.^[14,15]^ When lesions are extensive, staged ablations are allowed at the physician’s discretion. The second procedure will usually be performed within 3 months.

#### 2.3.2. Follow-up biopsy at visits 3 and 6

The patient will be placed in the left lateral recumbent position, an anoscope will be inserted to magnify the anal canal for colposcopy, 3% acetic acid will be applied, and the aceto-white lesion surface, margins, and vascular abnormalities will be observed. Sextant biopsy samples will be collected in all directions, regardless of abnormal findings, to investigate local and metachronous recurrences.

#### 2.3.3. Adverse events

Potential adverse events related to electrocautery ablation include bleeding, textural changes at the targeted lesions, and mild-to-moderate pain for up to 2 weeks after the intervention. Potential rare adverse events (<1%) include heavy bleeding, stenosis, fissure, fistula, and abscess.^[5]^

All serious adverse events occurring between the time informed consent was signed and the end of the trial will be documented in the medical records and reported to the Clinical Research Review Committee.

### 2.4. Primary and secondary endpoints

The primary endpoint is local recurrence of grade 2 or 3 AIN 6 months after the procedure. The secondary end points are presented in Table 2.

**Table 2:** Secondary outcomes.

Objective	Endpoint	Rationale for the endpoint
To assess the efficacy of ablation therapy	Metachronous recurrence† of grade 2 or 3 anal intraepithelial neoplasia at 6 mo after the procedure	It is not a subjective endpoint
To assess safety after ablation therapy	Occurrences of adverse events	It is necessary to evaluate safety
To assess the efficacy of ablation therapy	Local recurrence of grade 2 or 3 anal intraepithelial neoplasia at 3 mo after the procedure	It is not a subjective endpoint
To assess the efficacy of ablation therapy	Metachronous recurrence of grade 2 or 3 anal intraepithelial neoplasia at 3 mo after the procedure	It is not a subjective endpoint

† A metachronous recurrence is defined as a recurrence outside of the ablative area.

### 2.5. Data collection and management

A case report form (CRF) will be prepared by appropriate and authorized individuals (investigators and physicians). Unless data recorded directly in the CRF are used as the source material, all data recorded in the CRF must be consistent with the original material. During the course of the study, the investigator will collect data at each visit. All study findings and documents will be kept strictly confidential, and patients will be identified only by their patient number and/or birth date, never by name. To protect participant anonymity, the investigator will keep patient-identifying documents confidential.

The following data will be collected.

- Date of birth (age), sex, nationality, race, prior medical history, history of current disease, complications, allergy, and pregnancy status;
- Body weight and height at the first visit;
- Vital signs at every visit;
- Level of consciousness;
- Body temperature (°C);
- Blood pressure (mm Hg);
- Pulse rate (beats/min);
- Respiratory rate; and
- Physical condition at every visit (Physical conditions will be examined by inspection, palpation, auscultation, and percussion.).

### 2.6. Statistical analysis plan

The population to be analyzed is defined as all patients receiving electrocautery ablation. The characteristics of the study participants will be summarized using descriptive statistics. The recurrence rate at each visit will be estimated using point estimates and 95% confidence intervals. The safety analyses will be based on this analysis set. Adverse events will be stratified according to the patients’ HIV status and severity. Analyses will be categorized and aggregated by patient ID, and a list of outcomes will be generated. Data will be analyzed with STATA, version 16.0, and the sample size calculation will be determined using PASS 2022.

## 3. Discussion

The detection of AIN by screening is key to the prevention of anal cancer. Based on the similarity of cervical cancers, screening programs with interventions such as electrocautery ablation may be a favorable approach in high-risk populations. The ANCHOR study showed that interventions in patients with HSIL significantly reduced the risk of anal cancer. Electrocautery ablation is a promising intervention, particularly when the targeted area is extensive. Several studies have suggested high efficacy and low side effects.^[5,8,10]^ The major concern is the high rate of relapse, and according to previous studies, most recurrences occurred outside of the treated area.^[8,10]^ In our protocol and routine practice for HRA-guided biopsy, biopsy will be conducted in all directions regardless of HRA findings; thus, the results of this study will reflect an accurate clinical course based on pathological findings. This is the big difference between our study and other previous studies.^[5,8,10]^ Furthermore, in this study, the Surgitron dual EMC will be used for electrocautery, and the device has a high radiofrequency, which is expected to reduce postoperative discomfort, scar tissue formation, and enhanced healing while maintaining high efficacy.^[12]^

Several potential limitations of this study need to be acknowledged. First, the study is not a randomized controlled trial or single-arm trial. Therefore, the effectiveness of electrocautery ablation using the Surgitron dual EMC compared with another device is unknown. However, this study will evaluate safety first. Second, since this is a single-arm study with a small sample size, the result will likely be affected by selection biases. Third, the study population will include MSM with and without HIV. Other important at-risk populations include women with HPV-related cancer or precancerous lesions, people with immunosuppression, and people taking immunosuppressants who will not be included in this study. Further studies are needed to overcome these limitations.

## 4. Conclusion

In this study, we will treat HSIL patients with electrocautery ablation using the Surgitron dual EMC and follow-up with HRA-guided biopsy to determine the efficacy and safety of electrocautery ablation.

## Acknowledgments

We would like to thank Editage (www.editage.com) for English language editing and Yoshimi Deguchi and Mikiko Ogata, the study coordinators for their assistance in conducting this study.

## Author contributions

NA, DM conceived and designed the study. NA, DM, TA, TK, KW, HU, HG and SO contributed to data collection and curation. NA, DM, and MT were in charge of data curation and accessed and verified all data. HG and SO supervised. NA wrote the original draft of the manuscript. All authors contributed to refinement of and approved this manuscript. All authors had final responsibility for the decision to submit for publication.

**Conceptualization:** Naokatsu Ando, Daisuke Mizushima.

**Data curation:** Naokatsu Ando, Daisuke Mizushima, Misao Takano.

**Formal analysis:** Naokatsu Ando.

**Funding acquisition:** Naokatsu Ando.

**Investigation:** Naokatsu Ando, Daisuke Mizushima, Misao Takano, Hiroshi Kitamura, Daisuke Shiojiri, Takato Nakamoto, Takahiro Aoki, Koji Watanabe, Haruka Uemura, Hiroyuki Gatanaga, Shinichi Oka.

**Methodology:** Naokatsu Ando, Daisuke Mizushima.

**Project administration:** Naokatsu Ando, Daisuke Mizushima, Misao Takano.

**Resources:** Naokatsu Ando.

**Software:** Naokatsu Ando.

**Supervision:** Hiroyuki Gatanaga, Shinichi Oka.

**Validation:** Naokatsu Ando.

**Visualization:** Misao Takano.

**Writing – original draft:** Naokatsu Ando.

**Writing – review & editing:** Naokatsu Ando, Daisuke Mizushima, Misao Takano, Hiroshi Kitamura, Daisuke Shiojiri, Takato Nakamoto, Takahiro Aoki, Koji Watanabe, Haruka Uemura, Hiroyuki Gatanaga, Shinichi Oka.
